# Characterization of the genetic pool of the Canadienne dairy cattle breed

**DOI:** 10.1186/s12711-023-00793-3

**Published:** 2023-05-09

**Authors:** Alexandra Carrier, Isabelle Gilbert, Pierre Leclerc, Mario Duchesne, Claude Robert

**Affiliations:** 1 Centre de recherche en reproduction, développement et santé intergénérationnelle, Université Laval, Québec, QC Canada; 2 Institut sur la nutrition et les aliments fonctionnels, Université Laval, Québec, QC Canada; 3 Dé̇partement des sciences animales, Faculté des sciences de l’agriculture et de l’alimentation, Université Laval, Québec, QC Canada; 4 Association de Mise en Valeur de La Race Bovine Canadienne (AVRBC), Québec, QC Canada; 5 Dé̇partement d’obstétrique, gynécologie et reproduction, Faculté de médecine, Université Laval, QC Québec, Canada

## Abstract

**Background:**

Canadienne cattle are the oldest breed of dairy cattle in North America. The Canadienne breed originates from cattle that were brought to America by the mid-seventeenth century French settlers. The herd book was established in 1886 and the current breed characteristics include dark coat color, small size compared to the modern Holstein breed, and overall rusticity shaped by the harsh environmental conditions that were prevalent during the settlement of North America. The Canadienne breed is an invaluable genetic resource due to its high resilience, longevity and fertility. However, it is heavily threatened with a current herd limited to an estimated 1200 registered animals, of which less than 300 are fullblood. To date, no effort has been made to document the genetic pool of this heritage breed in order to preserve it.

**Results:**

In this project, we used genomic data, which allow a precise description of the genetic makeup of a population, to provide valuable information on the genetic diversity of this heritage breed and suggest management options for its long-term viability. Using a panel that includes 640,000 single nucleotide polymorphisms (SNPs), we genotyped 190 animals grouped into six purity ranges. Unsupervised clustering analyses revealed three genetically distinct groups among those with the higher levels of purity. The observed heterozygosity was higher than expected even in the 100% purebreds. Comparison with Holstein genotypes showed significantly shorter runs of homozygosity for the Canadienne breed, which was unexpected due to the high inbreeding value calculated from pedigree data.

**Conclusions:**

Overall, our data indicate that the fullblood gene pool of the Canadienne breed is more diversified than expected and that bloodline management could promote breed sustainability. In its current state, the Canadienne is not a dead-end breed but remains highly vulnerable due to its small population size.

## Background

In livestock management, genetic diversity is an important element to consider. To provide options for long-term breed improvement or the selection of animals for specific immediate purposes, diversity must be maintained by careful planning. Traditionally driven by profit margins, today dairy cattle breeding takes consumer expectations regarding product quality or safety and animal wellbeing increasingly into account. This often introduces new selection goals that challenge current genetic lineages, for example, adaptability to climate change, lower dependency on antibiotic usage, lower feed consumption (and hence farmland use for animal feedstuff production) and decreasing the overall environmental footprint of animal husbandry. Achieving such goals involves major changes to practices and to the environmental conditions to which the animals are subjected. Tolerating wider ranges of temperature, more variable diets, emerging pathogens and different husbandry methods will all require genetic adaptation. Heritage dairy breeds that have fallen into disuse due to their lower milk yield or productivity may be pressed back into service, since they often exhibit higher resilience and adaptive capacity. The Canadienne is one of these heritage cattle breeds. It is known for its high fertility and its remarkable calving ability [1]. Compared to other dairy breeds used in Canada, it is frugal, less affected by poor diet and environmental dynamics, although this can sometimes become problematic for the Canadienne at drying since it produces a small volume of milk even under restricted feeding, i.e. its annual yield is about 6000 kg [2]. Dairy producers currently use the Canadienne breed to serve specialty markets such as for the production of fine cheeses.

The origins of this breed date back to the first French settlements in North America. The first cattle were brought from the Bretagne and Normandy regions of France in the mid-seventeenth century. They were multi-purpose, providing milk and meat and pulling carts and ploughs. In the seventeenth and eighteenth century Canada, cattle had to be robust, autonomous and capable of surviving neglect (MacLachlan, personal communication). The Canadienne cattle developed high strength, strong resistance to the cold and to disease, and thrived on whatever it was given or could find in the fields. Up to the end of the nineteenth century, the Canadienne was most representative of the dairy cattle in New France and later in the Canadian province of Quebec.

The breed was officially recognized in 1886 and then followed a development through selective breeding mainly to improve milk production. However, European breeds soon began to gradually replace Canadienne cattle, and by the 1970s, only about 5000 registered Canadienne individuals remained. In an attempt to revitalize the breed, it was crossed with the Brown Swiss to improve milk production, stature and frame. In more recent years, Canadienne individuals have been mostly crossbred with Jersey to preserve a smaller stature and frame compared to other dairy breeds as well as to maintain their uniform coat color. Current herdbook registration lists animals that were never crossed and identifies them as “originals” and about 300 such full-bloods are alive today. Based on pedigree data, the estimated effective size of the Canadienne population dropped down to 43 in 2013 [3] and the inbreeding index was estimated at 9.16% in 2020 [4], thus the long-term survival of the breed is in jeopardy. The Livestock Conservancy lists its status as critical [5].

The hypothesis of our research project stipulates that the use of genomics will enable to properly describe the Canadienne genetic pool to offer long-term management options. The aims of the project are to describe the genetic pool of the Canadienne cow using high-density genotyping and to use the information to determine if the low effective size has pushed the breed to a genetic dead-end. Current genotyping platforms targeting large panels of known single nucleotide polymorphisms (SNPs) allow an efficient description and comparison of mammalian genomes. In this work, we used genotypes to generate diversity metrics to compare groups of animals with different percentages of Canadienne blood. The analyses included evaluation of its population structure and genomic inbreeding and comparisons with other bovine breeds and populations.

## Methods

### DNA sampling

Pedigrees and the Canadienne Cattle Association and Society were consulted to obtain a representative sample of the population and different levels of genomic diversity and purity. The Canadienne population was sampled in 2016 from 10 herds located across the Province of Quebec. A small herd maintained in France was also sampled and a bull living in the USA was included. The founders of these foreign animals were imported from Quebec in the 1990s. DNA was extracted from tail hair follicles. Cryopreserved semen from ancestral bulls that lived from 1950 to 1980 were obtained from an insemination center (Centre d’insémination artificielle du Québec, CIAQ) and were also genotyped. Based on breed purity, six genomic groups were formed (Table 1).

**Table 1:** Canadienne cattle groupings based on genetic purity

Group	Genetic purity range, %	Breed fraction	Sample size
Original	100	32/32	36
Fullblood (Pureblood)	93.75–100	15/16–31/32	77
High purity	87.5–93.74	7/8–15/16	31
Moderate purity	75–87.4	3/4–7/8	32
Crossed	50–74	1/2–3/4	11
Low purity	0–49	< 1/2	3

To compare the genetic diversity of the Canadienne with that of other breeds, the genotypes of 192 Canadian Holstein bulls born between 2007 and 2012 were accessed from a database. Semen was obtained from genetic companies operating in North America. This previous project was supported by a grant from Novalait as a pilot project to assess the genetic diversity in the Holstein population in Canada. SNP data from eight other breeds (Angus, Bretonne Pie Noir, French Brown Swiss, Brown Swiss, Guernsey, French Holstein, French Jersey, Normande) were obtained from previous studies [6, 7]. *Bos indicus* SNP data from a previous study [8] were included as an outgroup. Breed representation is provided in Table 2.

**Table 2:** Breed representation in each genotypic dataset

Breed	n	Source
Angus	62	[9]
Bretonne Pie Noir	18	[7]
French Brown Swiss	18	[7]
Brown Swiss	24	[8]
Guernsey	21	[8]
French Holstein^a^	93	[7, 8]
French Jersey	49	[7, 8]
Normande	30	[7]
Zebu Bororo	23	[9]
Arabic Zebu	35	[9]
Zebu Fulani	43	[9]

^a^BovineSNP50v1 genotyping platform

### DNA extraction

Among the 190 Canadienne DNA samples, 62 were extracted from cryopreserved semen and 128 from tail hair. All the samples were stored at − 20 °C until analysis. Two samples were analyzed in duplicate for SNP genotyping quality control. Genomic DNA was extracted from bovine spermatozoa using the DNeasy blood and tissue kit (QIAgen, Toronto, ON, Canada) and following the user-developed protocol 2 for purification of total DNA from sperm. Briefly, the entire contents of one semen straw or vial (~ 12–15 × 10^6^ cells) were mixed gently with one mL of phosphate-buffered saline (pH 7.4) in a 1.5-mL microcentrifuge tube, and then centrifuged at 15,000*g* for five min. The supernatant was discarded, and the sperm pellet (100 µL) was mixed with 100 µL of X2 buffer (20 mM Tris pH 8.0, 20 mM EDTA, 200 mM NaCl, 4% SDS and 80 mM dithiothreitol) and 12.5 µL of the kit proteinase K solution. The suspension was held overnight (12–16 h) at 56 °C in a hybrization incubator (Robbins Scientific, San Diego, CA, USA) with rotation set at 12 rpm, then mixed with 200 µL of buffer AL (QIAgen) and 200 µL of ethanol (96–100%), loaded onto the spin column (QIAgen) and centrifuged for one min at > 6000* g*. Column-bound DNA was washed and eluted according to the manufacturer’s instructions.

DNA was extracted from hair follicles according to the user-developed protocol for purification of total DNA from nails, hair or feathers using the DNeasy blood and tissue kit (QIAgen). Briefly, 20 to 30 tail hair follicles with 0.5–1.0 cm of bristle were placed in a 1.5-mL microtube. A lysis buffer containing 300 µL of ATL buffer, 20 µL of proteinase K and 20 µL of 1 M dithiothreitol (QIAgen) was added and the contents were mixed by pulse-vortexing, held overnight at 56 °C in the hybridization incubator set at 20 rpm, treated with RNase A (4 µL, 100 mg/mL, QIAgen) for 2 min at room temperature then mixed with 300 µL of AL buffer (QIAgen) and 300 µL of ethanol (96–100%), loaded onto a spin column (QIAgen) and centrifuged for 1 min at > 6000*g*. DNA was washed and eluted according to the manufacturer’s protocol.

Genomic DNA quality was assessed by electrophoresis on 1% agarose gels and staining with ethidium bromide (1 µg/mL). DNA was quantitated by measuring the absorbance at 260 nm using a NanoDrop1000 (ThermoFisher Scientific, Ottawa, ON, Canada).

### SNP genotyping and SNP datasets for comparative purposes

In total, 192 Canadienne samples (two of the 190 animals were genotyped twice as quality control replicates) were genotyped using the Affymetrix/ThermoFisher Axiom BOS1 platform targeting 648,874 SNPs. Canadian Holstein genotypes were acquired within the context of a different project on the Illumina Bovine SNP50 v3 platform targeting 53,218 SNPs. All sample hybridizations were performed at the Centre d’expertise et de services Génome Québec (Montréal, QC, Canada).

### Sample exclusion, quality control and SNP filtering

Quality control and SNP filtering were carried out using the Axiom Analysis Suite. SNPs with a call rate lower than 97% across all Canadienne samples were excluded. Monomorphic SNPs and those with a minor allele frequency lower than 0.05 were also excluded because due to their very low statistical power, they are not useful for analyses comprising only the Canadienne breed. Significant departure from the Hardy–Weinberg equilibrium (HWE) was also considered since it can indicate potential genotyping errors. Thus, SNPs with a HWE FDR/Bonferroni corrected p-value lower than 1e-3 were filtered out. Only autosomal SNPs with a DishQC score ≥ 0.82 and a call rate ≥ 97% were retained for further analysis.

### Identification of common SNPs between genotyping platforms

Since the Thermo Fisher-Affymetrix/and Illumina platforms were both developed using the SNP catalog generated from the bovine genome project, there is great commonality between the two panels. For the initial comparison of the Canadienne with the Canadian Holstein breed, a common set of SNPs shared by both genotyping platforms was identified by positioning targeted SNPs from both platforms on the same reference genome (UMD 3.1 *Bos taurus*). The same procedure, i.e. positioning all SNPs across all datasets on the same reference genome (UMD 3.1 *Bos taurus*) was performed to identify the SNPs that were common to all datasets for the across-breed and population comparisons. All common SNPs were kept and were not filtered for allelic frequencies. Allele flipping between both genotyping platforms was checked and for none of the SNPs was the reference allele considered as the alternate allele in the other platform. About half of the SNPs were found to be detected on opposite strands (24,990/49,397) but this does not impact the analyses since genotypes do not report the nucleotides but the status of both alleles, i.e. *AA*, *AB* or *BB*.

### Genetic metrics

Observed heterozygosity (H_O_) was evaluated using the—het option in VCFtools v0.1.11 and genome-wide SNP data. Genomic inbreeding (*F*_ROH_) was evaluated using runs of homozygosity (ROH) with the—lroh option and expressed as the *F*_ROH_ statistic, that is, the ratio of the total ROH length (*L*_ROH_) to the total autosomal genome length covered by the SNPs (*L*_GENOME_) [9]:

$${F}_{\mathrm{ROH}}=\frac{\Sigma {L}_{\mathrm{ROH}}}{{L}_{\mathrm{GENOME}}}.$$

The genomic effective population size (N_e_) was estimated using the SNP excess heterozygosity method implemented by the NeEstimator program [10]. The per site heterozygosity was evaluated using the –hardy option in VCFtools v0.1.11 with reduced datasets matching the Canadienne and Holstein genotypes. Two comparisons were performed: (1) between the registered Canadienne individuals and all the Canadian Holsteins and (2) between only the 36 “original” Canadienne individuals and equivalent number of randomly selected Canadian Holsteins. One hundred subsets of 36 Canadian Holsteins were randomly sampled using the –max-indv filter, and the mean per site H_O_ was calculated to match the number of “original” Canadienne individuals.

### Population structure

The VCFtools v0.1.11 program was used to compute the fixation index (F_ST_ statistic) using the—weir-fst-pop option. Individuals were grouped according to breed purity for comparison. To assess the genomic variation among the Canadienne populations, an unsupervised clustering using a discriminant analysis of principal components (DAPC) [11] was performed on genome-wide SNP data using the R function DAPC in the ADEGENET package [12].

### Across-breed comparison

To assess genomic variation among the 12 breeds/populations considered, principal component analysis was performed using the R function snpgdsPCA in the SNPRelate package [13]. Sample size was equilibrated. For datasets containing more than 40 samples, a randomized sampling was done for 40 samples. For datasets containing less than 40 samples, all samples were used. The French Brown Swiss and Brown Swiss datasets were merged (Table 2).

### Statistics

Significant differences between the purity group means were estimated for H_O_, *F*_ROH_ and ROH length classes using Duncan’s test in the agricolae R package [14] with the function duncan.test.

## Results

### Data processing and selection of SNP panels

For the Canadienne samples genotyped on the high-density panel (640K), 634,116 high-quality SNPs for 190 animals remained after filtering. For the 192 Holsteins genotyped on the medium-density panel (50K), 48,725 acceptable SNPs were obtained and 47,573 remained after selecting the common target loci between both SNP panels. When public data from the nine other breeds/populations were considered, 47,175 high-quality SNPs were found across all datasets.

### Genetic diversity in the Canadienne population

**Fig. 1:**
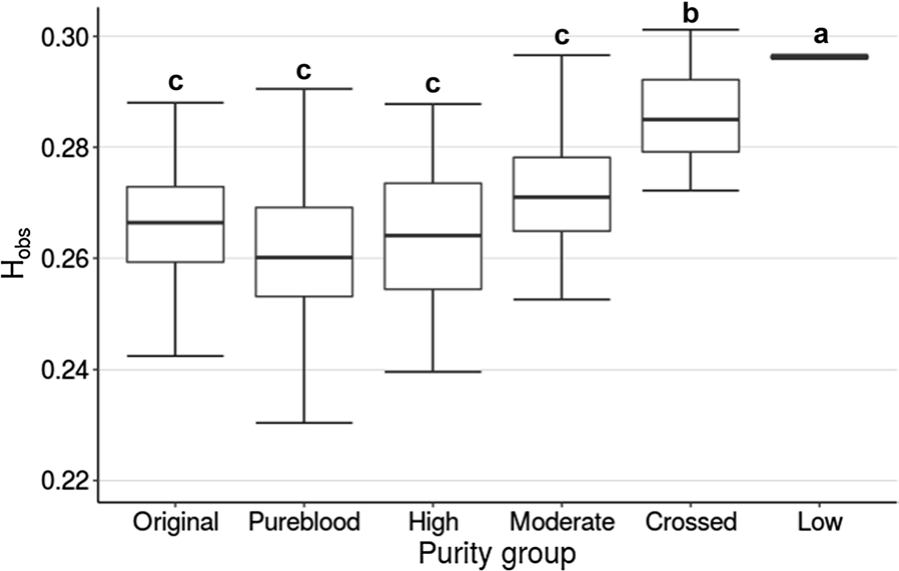
Distribution of observed genetic heterozygosity (H_o_) in each Canadienne cattle breed purity group. Boxes represent second and third quartiles; letters indicate a significant difference, based on Duncan’s test (P < 0.05)

Genome-wide heterozygosity was calculated for each animal and ranged from 0.2201 to 0.3011 with a mean of 0.2660. The mean for each group is shown in Fig. 1. The genome-wide heterozygosity for the original, fullblood (Pureblood) and high-purity Canadienne groups did not significantly differ, which indicates that, above a breed fraction of 3/4, the extent of heterozygosity was similar even in the “original” Canadienne group. As expected, crossed and low-purity groups had a significantly higher H_O_ whereas moderate-purity individuals had intermediate values (Fig. 1).

**Fig. 2:**
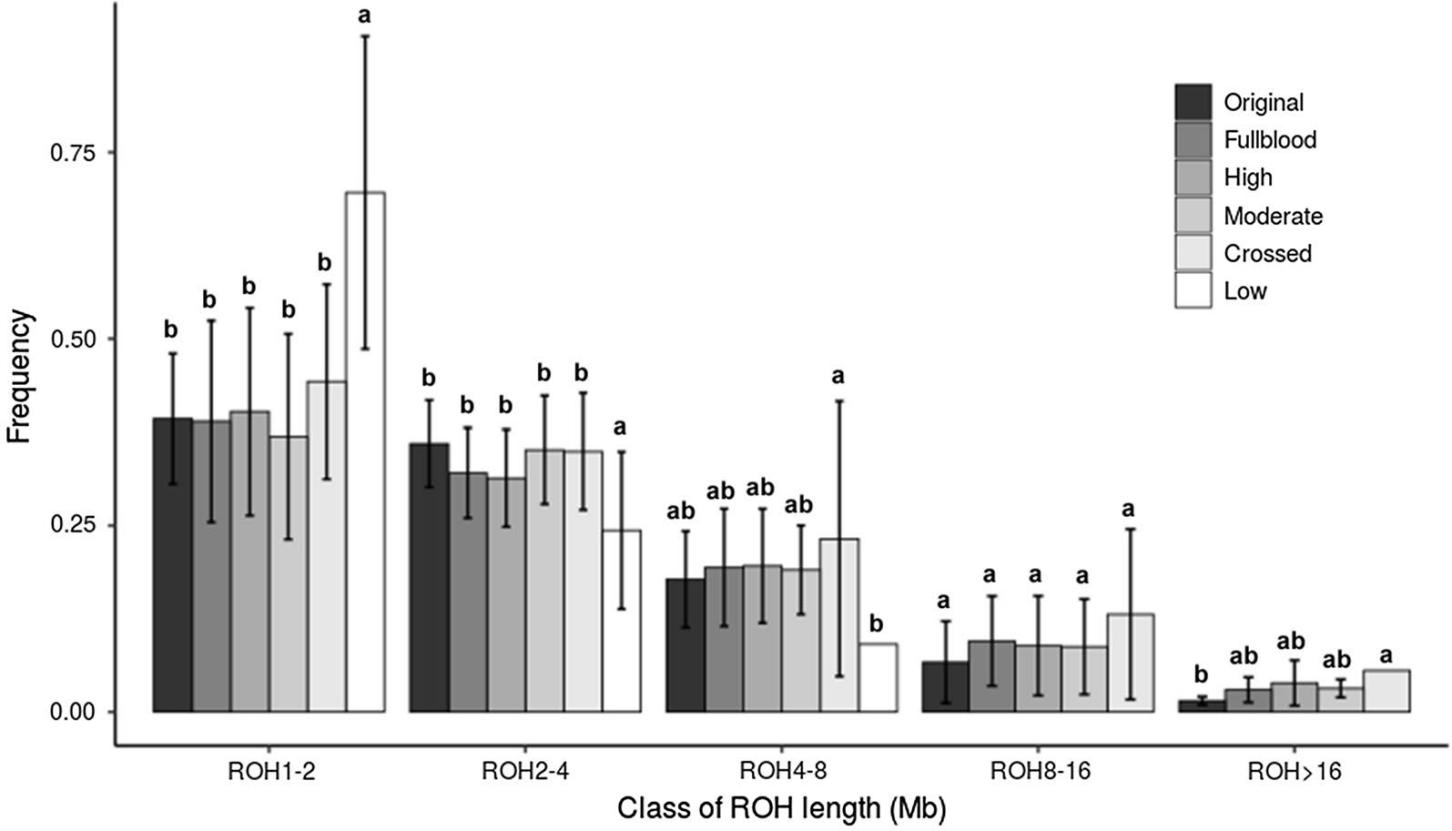
Frequency of the five runs of homozygosity (ROH) length ranges (in Mb) in the six Canadienne purity groups, based on genome-wide analysis of 640K SNPs. Standard deviation is shown as error bars (low purity at the ROH range of 4–8 Mb contained a single individual). Letters indicate a significant difference among purity groups for each ROH range, based on Duncan’s test (P < 0.05). Darkest to lightest shade: original, fullblood, high purity, moderate purity, crossed, low purity

The lengths of the genomic stretches with homozygous genotypes were measured as another metric of genomic diversity. Runs of homozygosity were classified by length, with the shorter ROH indicating greater genetic diversity. The frequency of each length class measured for individual animals and the mean frequencies for each purity group are presented in Fig. 2. As expected, mixed-breed animals with less than 50% Canadienne lineage had the highest proportion of shorter (1–2 Mb) ROH. The high-purity groups, namely the original (100%), fullblood (> 15/16), high and moderate-purity groups, had similar proportions of short and long ROH (Fig. 2).

**Fig. 3:**
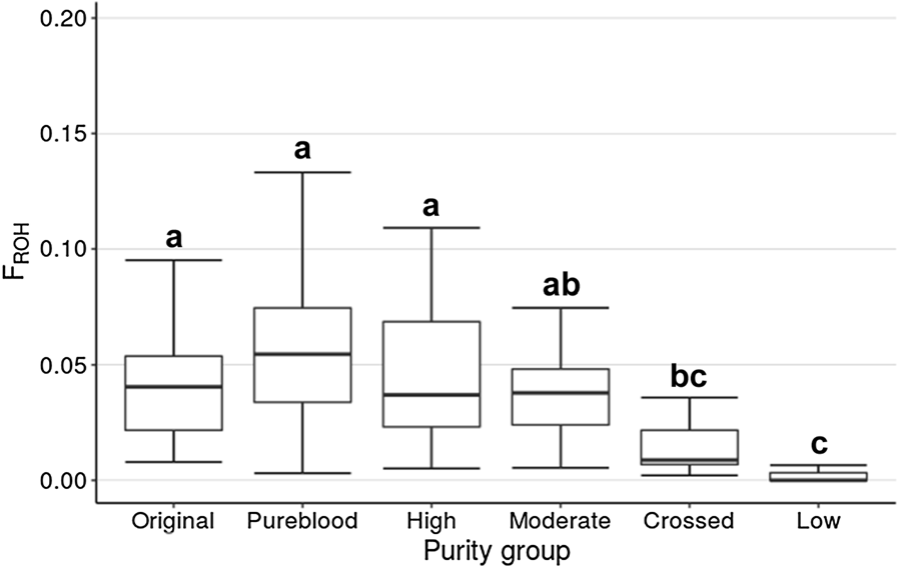
Comparison of individual inbreeding values in the Canadienne purity classes, based on ROH lengths (*F*_ROH_). Boxes represent class median and second and third quartiles. Letters indicate a significant difference, based on Duncan’s test (P < 0.05)

The genomic inbreeding coefficient (*F*_ROH_) estimated for each animal ranged from 0 to 0.197. As expected, crossbred animals had very low inbreeding values (Fig. 3). The mean value for the combined classes corresponding to registered animals (> 7/8 Canadienne) was 0.051. Calculated for purity classes, the genomic inbreeding mean values for the original, fullblood and high-purity individuals were 0.044, 0.056 and 0.049, respectively. Again, no significant differences were found between groups from the original down to the moderate levels of purity (Fig. 3).

### Population structure of the Canadienne breed

#### Effective population size

The effective population size was estimated for each purity group and averaged for the population composed of animals with at least 3/4 Canadienne lineage (Table 3), and it was low in the “originals” group (12.6) and higher in the fullblood (20.5) and high-purity groups (34.9).

**Table 3:** Estimated effective population size for each Canadienne breed purity group

Population	Sample size	Genomic N_e_	95% confidence interval limits
Original	36	12.8	12.6–12.9
Fullblood (Pureblood)	77	20.5	20.2–20.8
High purity	31	34.9	33.5–36.3
Moderate purity	32	12.9	12.7–13.0
Total	176	20.8	20.6–21.0

#### Genetic distance

The mean F_ST_ estimated from genotypes was used to compare each pair of purity groups and ranged from 0.0014 to 0.0700 with an average value of 0.0385 (Table 4). It was lowest for the original versus fullblood comparison and highest for the original versus crossed individuals comparison. As expected, F_ST_ increased as the difference in purity increased.

**Table 4:** Pairwise *F*_ST_ between Canadienne breed purity groups

Purity group	Original	Fullblood	High	Moderate
Fullblood (Pureblood)	0.0014			
High purity	0.0258	0.0176		
Moderate purity	0.0329	0.0278	0.0238	
Crossed	0.0700	0.0580	0.0605	0.0559

#### Clustering

**Fig. 4:**
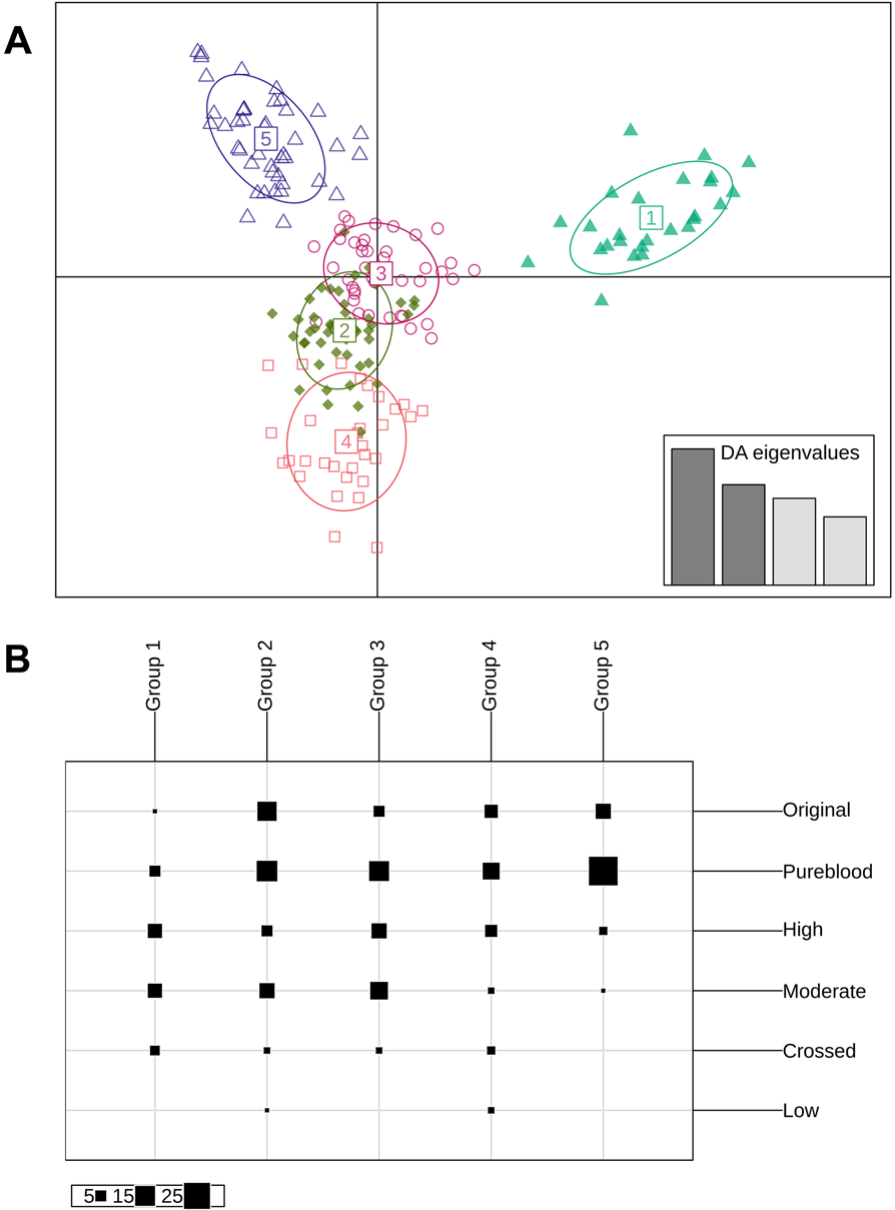
**a** Discriminant analysis of principal components (DAPC) showing five clusters with a low degree of overlap among individuals from the Canadienne cattle population sample. Inset indicates the number and contribution of the axes from the principal components analysis retained in the discriminant analysis. **b** Contingency table of the purity groups *vs* the inferred groups by DAPC. Columns correspond to inferred clusters and rows to purity groups. The size of the squares represents the number of individuals

Unsupervised clustering of genotypes revealed five intrapopulation clusters (Fig. 4a), each of which contained animals from groups with different levels of purity, as shown in the contingency table (Fig. 4b). Cluster 5 was mostly enriched in individuals at the higher end of the purity scale whereas cluster 2 contained animals of very high purity (original) as well as lower purity classes. Clusters 3 and 4 were composed of a distribution across purity classes whereas clusters 1 contained mostly low-purity individuals (Fig. 4b).

#### Across-breed comparison

**Fig. 5:**
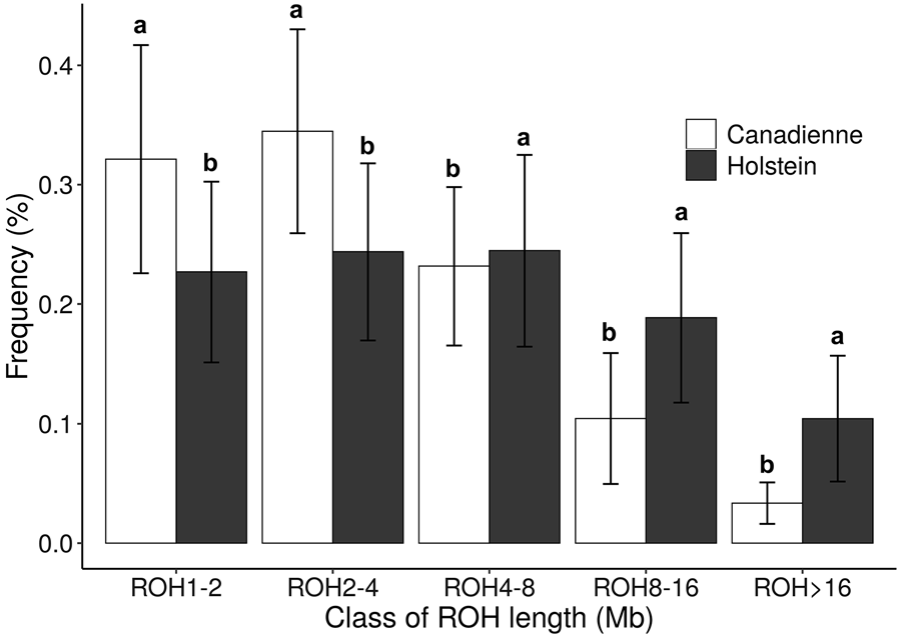
Frequency of the five ROH length ranges (in Mb) in the Canadienne (at least 7/8 purity) and Canadian Holstein breeds, based on genome-wide analysis of 50K SNPs. Standard deviation is shown as error bars. Letters indicate a significant difference between breeds for each ROH range, based on Duncan’s test (P < 0.05)

Using the reduced dataset composed of the common SNPs between the high- and medium-density SNP panels, ROH lengths were recalculated for the Canadienne breed and compared with Canadian Holsteins, which had a significantly higher proportion of longer ROH and a lower proportion of shorter ROH (Fig. 5). This reflects the lower genetic diversity and recent inbreeding events that have occurred in this breed.

**Fig. 6:**
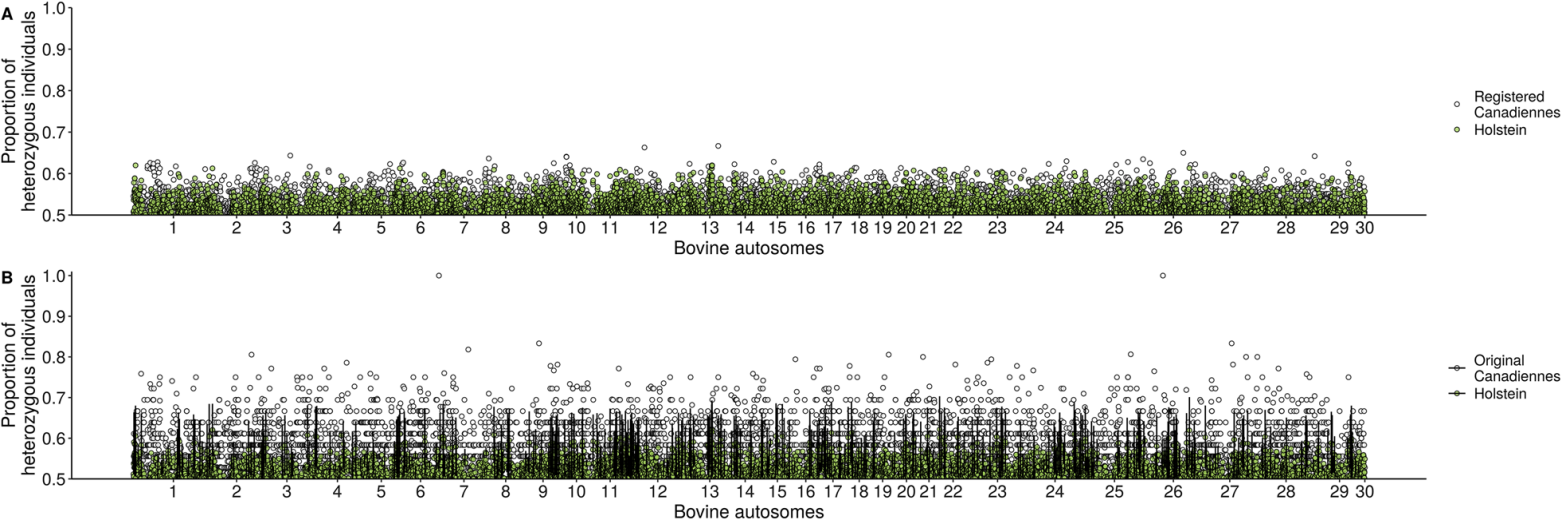
Genotypic frequencies of heterozygotes at individual loci on bovine autosomes. **a** Comparing registered Canadienne (n = 176) (at least 7/8 purity) and Holsteins (n = 192) and **b** Comparing Original Canadienne (n = 36) and a group of Holstein (n = 36). Holstein values represent the mean of 100 random resampling. Standard deviations for Holstein are shown

The per site H_O_ ranged from 0 to 0.667 for the registered Canadienne (> 7/8 Canadienne), from 0 to 0.620 for the Canadian Holstein, and from 0 to 1 for the “original” Canadienne individuals (Fig. 6). For most of the sites, H_O_ was higher for the Canadienne (registered and “originals”) than for the Canadian Holstein individuals.

**Fig. 7:**
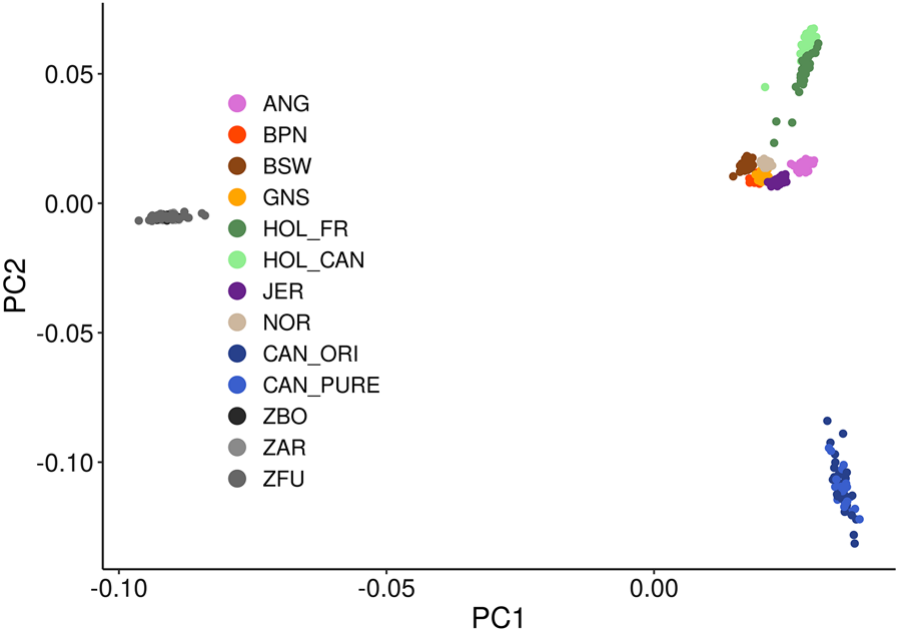
Principal component analysis plot comparing different bovine breeds and populations. *ANG* Angus, *BPN* Bretonne Pie Noir, *BSW* Brown Swiss, *GNS* Guernsey, *HOL_FR* French Holstein, *HOL_CAN* Canadian Holstein, *JER* French Jersey, *NOR* Normande, *ZBO* Zebu Bororo, *ZAR* Arabic Zebu, *ZFU* Zebu Fulani, *CAN_ORI* Original, *CAN_PURE* fullblood

Unsupervised clustering using genome-wide SNP data showed inter-population diversity and the genetic relationships across cattle breeds. The first and second principal components (PC1 and PC2) are plotted in Fig. 7. For this PCA analysis, about 40 samples per breed were used (see Methods) and four groups of Canadienne are represented according to level of purity. The PC1 axis places *Bos taurus* breeds at the opposite end of *Bos indicus* cattle breeds. On the PC2 axis, Canadienne cluster away from the other breeds. On PC2, both Canadian and French Holsteins are further away from the other assessed breeds. The closest cluster is Jersey, a breed that has recently been crossed with Canadienne. The Canadienne clustering is not as tightly centered as many other purebred gene pools indicative of more diversity.

## Discussion

The goal of this project was to describe the genetic makeup of the Canadienne bovine breed based on genomics. This breed has declined from 300,000 individuals in the early 1900s to about 10,000 in 1983 and then to 1000 in 2012 with less than 300 full blooded animals [15, 16]. There are concerns about the long-term viability of the breed. Based on pedigree data, the Canadienne has the highest inbreeding coefficient of any dairy cattle breed in Canada, and an average inbreeding coefficient of 9.53% as calculated in 2022. By comparison, the mean inbreeding coefficients of the Canadian Holstein and Jersey breeds are, respectively, 8.86 and 7.10% [4]. Its low milk production prevents the Canadienne breed from being a viable alternative to the more common breeds for commercial bulk milk under the quota system that is in place in Canada, and its high inbreeding coefficient deters dairy producers from adding Canadienne cows to their herd. In addition, the decision to improve the breed by crossing it with Brown Swiss in the 1970s followed by backcrossing with Canadienne has raised doubts about its retention of the original gene pool shaped by the harsh conditions that they encountered during the European colonial period in North America. The registration requirement for purebred animals with all-Canadienne ancestry (the “originals”) has further reduced the number of animals in the highest purity class.

Our objective was to use genomics to estimate genetic diversity and to determine if the inbreeding coefficients based on pedigree data would increase when the population was segmented according to purity class. To maintain the “original '' status, an animal must be the offspring of a dam and sire that are also “original”. The breed currently relies on artificial insemination limited to about 15 sires of which only two are purebred “originals”. To maintain this status, producers can raise their own bulls or use cryopreserved semen collected prior to 1980. With such constraints on sires, it was expected that the inbreeding coefficient would be higher for “originals'' than for the breed. However, the genetic diversity of this purity class was comparable to that of classes down to 7/8 Canadienne. This was inferred from the extent of heterozygous genotypes and the lengths of homozygous genomic stretches. At 6.8%, the calculated genomic inbreeding coefficient is lower than the reported 9.65% [4]. By comparison, a very comprehensive analysis done on 676,594 Holstein born in Canada between 1990 and 2018 showed that compared to the 2018 pedigree-based inbreeding estimate of 7.74%, genomic inbreeding values ranged from 13.61 to 15.64% [17]. Our observation of longer ROH in Holstein is consistent with these findings.

Although both pedigree- and genomic-based values are used to estimate the probability of receiving identical alleles from common ancestors, SNP analysis and pedigrees differ considerably. Pedigrees are subject to human error such as misidentification of the sire [18] and therefore may not match genomic data. More importantly, random processes of segregation and recombination lead to different genomes for individuals from the same pedigree unless they are identical twins. When a sufficient number of genomic markers (e.g., SNPs) is available, estimates of inbreeding are more accurate than those obtained from pedigree data [19, 20].

Runs of homozygosity are caused either by a high degree of relatedness or intensive selection for specific traits. Studies have shown that the proportion of ROH in the genome is a good proxy for autozygosity, i.e. superior to pedigree data [9, 21], and that the inbreeding coefficient increases with ROH length [22]. Longer ROH are due to recent inbreeding while shorter ROH reflect repeated meiotic events from ancient matings [23]. We compared the Canadienne genotypes to those of Holstein, the latter being the most common dairy breed in Canada, accounting for 93% of the 1.4 million animals in the national herd [2]. Holstein genetics benefit from extensive international trading, making it the reference point for Canadian dairy breeds. ROH lengths differed greatly between the two breeds. Holstein had an unexpectedly higher proportion of longer ROH than Canadienne cattle, especially of ROH longer than 16 Mb. The proportion of heterozygotes per site also demonstrated higher diversity in the “Original” and registered Canadienne groups than the Canadian Holsteins. This loss of heterozygosity in US Holsteins cattle results from the strong genetic selection for milk production and is associated with fertility and immunity genes [24], which is not an issue with the Canadienne breed as stated above. Genomic selection is known to have induced an increase in the number of ROH in North-American Holsteins [25], which are linked with fertility issues in this breed [26].

For all dairy breeds, inbreeding coefficients tend to increase due to limited numbers of sires and shorter generation intervals [27]. Genealogical surveys show that the development of the Holstein populations depends on a few sires, each of which now have millions of descendants [28]. The yearly rate of Holstein inbreeding has increased significantly since the introduction of genomic breeding programs [25]. Based on ROH, the impact of recent inbreeding events on Canadian Holstein genetics is considerable. Such events are not perceptible in the small Canadienne population, which is probably due to planned sire and lineage rotations in response to the awareness of the very limited number of sires. In addition, the Canadienne is a relatively recent breed that descends from mixed animals and thus has a more diversified gene pool to start with. Overall, our data strongly suggest that the Canadienne genetic pool is still diversified and that inbreeding expressed as the accumulation of homozygous genotypes is lower than anticipated.

For breeding purposes, the effective population size (N_e_) is considered critical when it drops below 50 individuals [29]. Even large herds can suffer from low N_e_ when the number of progenitors is small. Intense selective pressure can lead to the use of a small number of elite bull progenitors, especially when artificial insemination is highly accessible, and the current objectives to respond to consumer preferences and market trends can limit the number of sires that actually contribute to the next generation. Thus, generations of top-rated bulls may end up sharing ancestry [30]. The effective population size can fluctuate greatly depending on sire usage. During the 1990s, it dropped to a record small number of 33 in the Canadian Holstein herd, which is mainly due to the use of the famous Hanoverhill Starbuck bull that sired over 200,000 female offspring and 209 proven male offspring from about 1985 to 1995 [28]. During the period between 2000 and 2007, the N_e_ rose to 114 through diversification of sire provenance and usage.

Estimates of N_e_ based on pedigree data were equal to 54, 47, 46, 66 and 40, for Ayrshire, Brown Swiss, Guernsey, Milking Shorthorn and Canadienne, respectively [3]. Based on genotypes, a comprehensive analysis of the Holstein and Jersey populations in Canada found similar figures with N_e_ averaging 51 and 75, respectively, across estimation methods [17]. In our study, the N_e_ of the Canadienne was below 26.3 for purity classes > 3/4 and 12.8 for originals. This is alarming considering that the Canadienne gene pool does not exist in any other bovine population. One potential solution would be to increase the number of bulls available for artificial insemination, since currently there are only two purebred bulls at a local insemination center. Cryopreserved semen and embryos from genetic lineages less represented in the current herd could be used to generate new sire lines.

Analysis of the population structure showed that there are three Canadienne lineages enriched with high-purity animals. Genetic clusters are expected when different breeds are compared, but such genetic groups can also occur within breeds when high-density genomic markers are used, as seen in parentage assignment and pedigree reconstruction [31]. Pedigree-based data for individuals within each group show that inferred genetic clusters correspond to distinct ancestors, and that additional sire lines could only be beneficial. In such cases, crossing individuals from mixed family groups would increase genetic diversity and thereby reduce the impact of inbreeding [32]. Future breeding programs could prioritize crossing sires and dams from the most distant clusters to minimize genomic relatedness and thereby reduce the loss of genetic diversity and maintain N_e_.

Regardless of genetic management, the long-term survival of any gene pool depends on a sustainable and increased use of the breed. Yearly milk production is currently lower for Canadienne cows than for any other dairy breed in Canada [2] and improving it with such a low N_e_ is a considerable challenge. The solution may reside in the uniqueness of the gene pool of the Canadienne breed, since comparative genomic analysis has shown that it clusters independently of the other breeds. This could justify not only its conservation but also further analyses that may reveal unique and important traits for producing milk in different settings.

In our study, the Holstein individuals were also genetically isolated, which is probably due to the extensive selection programs for dairy production traits and conformity. Holstein cows are known to produce large volumes of milk but also to suffer from fragile health and lower fertility [24], which contrasts with the Canadienne cows characterized by their low milk production and high fertility. This could explain the genomic distance between Holstein and the other breeds. Moreover, the homogeneity of the other breeds compared to the Canadienne breed could be due to the absence of breeding programs in the latter case, since most of the current breeding programs aim at selecting the same economically important traits, which may reduce genetic variation and increase genomic homogeneity [33].

Since the Canadienne breed was developed mainly in Quebec, it is regarded as cultural patrimony, and in 1999, the provincial government claimed the designation “Québec heritage breed” [34]. Given the small size of the herds for this breed, conserving the cultural phenotype and improving animal usefulness may be conflicting objectives. Although its low dairy productivity prevents milk producers to adopt it, producers for specialty markets such as cheese products appreciate the cheese-making properties of its milk, and also its ability to adapt to different pastures could contribute to cheese uniqueness. Breeds of lower economic value can offer unique genetic characteristics that may benefit to commercial breeds [35]. By making suitable tradeoffs, it may be possible to define mutually beneficial goals for breed development and conservation that will add value to the dairy products sold [36] and will help endangered or threatened livestock breeds.

## Conclusions

Based on the use of genomic data, we demonstrate that the current effective population size of the Canadienne breed is alarming due to the decline in its popularity during the last century. The need for a wider selection of sires is urgent. In spite of the limited population and the constraining registration criteria for “original” status, its genetic diversity remains acceptable, and signs of recent inbreeding events are fewer than those observed in large population breeds under intense commercial selection pressure. To date, genomic-based selection programs have not been attempted to improve heritage breeds, and are not likely to be used because of the difficulties to establish a reference population necessary for genomic prediction and the small numbers of individuals in such populations [37]. However, genomics can assist in maintaining a healthy balance between selection and genetic variation within a livestock herd.

## Data Availability

The datasets used and/or analyzed during the current study are available from the corresponding author on reasonable request.
